# The role of life context and self‐defined well‐being in the outcomes that matter to people with a diagnosis of schizophrenia

**DOI:** 10.1111/hex.12548

**Published:** 2017-04-03

**Authors:** Helen Lloyd, Joanne Lloyd, Ray Fitzpatrick, Michele Peters

**Affiliations:** ^1^ Peninsula Medical School Plymouth University Devon UK; ^2^ School of Psychology Sport and Exercise Staffordshire University Stoke on Trent UK; ^3^ Nuffield Department of Population Health University of Oxford Oxford UK

**Keywords:** goals, patient centred outcomes research, qualitative research, Schizophrenia

## Abstract

**Objective:**

Conduct a deep exploration of the outcomes that matter to people with a diagnosis of schizophrenia and understand from their perspective how these outcomes can be achieved.

**Sample and Methods:**

In‐depth qualitative interviews were conducted with 22 people with a diagnosis of schizophrenia. Interviews were analysed using thematic frameworks, and a realist informed theories of change approach.

**Results:**

Our study revealed the potential causal relationships between the context of a person's life, short‐term goals and long‐term outcomes. We provide a nuanced and detailed exploration of outcomes that matter for people with schizophrenia in relation to self‐defined well‐being. Achieving life milestones, feeling safe and outcomes related to improved physical health along with employment, a positive sense of self and psychosocial outcomes, were highly valued. For short‐ and long‐term outcomes to be achieved, individuals required medication with minimal side‐effects, cognitive behavioural therapy, family/social support and meaningful activities in their lives. Well‐being was influenced by life context and short‐ and long‐term outcomes, but in a circular nature also framed what short‐term goals could be achieved.

**Conclusions:**

Working with people with a diagnosis of schizophrenia to identify and achieve better outcomes will necessitate a person‐centred approach. This will require an appreciation of the relationship between the statutory and non‐statutory resources that are available and a consideration of an individual's current well‐being status. This approach acknowledges personal strengths and encourages ownership of goals and supports self‐management.

## 1 INTRODUCTION

People with a diagnosis of schizophrenia often experience poor quality of life,^1^ require long‐term treatment^2^ and suffer from a range of physical health issues.^3^ Deficits in cognition^4^ and psychosocial and occupational functioning are common.^5^ There is also marked heterogeneity in how individuals experience the condition and their long‐term outcomes,^6^ which are affected by a range of factors across populations.^7^

In recognition of the considerable personal and societal impact of schizophrenia,^6^ improving outcomes is a core aim of clinical psychiatry and health policy.^8^, ^9^ Until recently, however, outcomes have been dominated by clinically defined improvement such as symptom reductions.^10^ Symptoms are commonly treated by medication, but response remains highly variable,^11^ and a significant proportion of people discontinue their use due to side‐effects or poor efficacy.^12^ Tensions between clinician and patient ratings of outcomes have also been reported, particularly in relation to the side‐effects of medication.^13^ People with a diagnosis of schizophrenia consider a broader range of outcomes as important,^10^, ^14^, ^15^ particularly those within the psychosocial domains (e.g. not only reductions in clinical symptoms).^10^, ^16^ Such outcomes are central to recovery‐based principles and more clearly aligned to person‐centred or collaborative care processes.^10^, ^15^ Recovery‐based processes work towards improvements in self‐direction and empowerment, providing holistic and individualized care to promote hope and respect. They emphasize and promote a strength‐based model and address interpersonal connectivity through peer support.^1^

A person‐centred approach to care could mitigate the misalignment described above by focusing on the outcomes that matter to people with schizophrenia. To date, few studies have sought to probe outcomes that matter to people with schizophrenia using in‐depth approaches. This knowledge would provide a conceptual and theoretical starting block for subsequent empirical research.

### 1.1 Aims of the study

The aim of this study was to conduct a depth exploration to identify the outcomes that matter to people with schizophrenia and to explore from their perspective the ways in which these could be achieved.

## 2 MATERIAL AND METHODS

### 2.1 Design

This qualitative study was part of a research project consisting of a literature review, stakeholder conference and a study with “self‐defined” carers of people with schizophrenia. Ethical approval was obtained from NHS East of Scotland Research Ethics Service (EoSRES) REC 1 (Application Number 13/ES/0143). Participants gave written consent to participate in the study.

### 2.2 Sample

Participants were sent an invitation through Rethink Mental Health and recruited by snowball sampling through family members who participated in our carer study. Sixty‐one people responded to the invitation. Thirteen people were not interviewed as they did not have a diagnosis of schizophrenia; a further five people refused to be interviewed. Interviews were conducted with twenty‐two people of a possible forty‐three who responded and were eligible. The decision to stop interviewing was determined by data saturation which was reached when no uniquely new themes emerged in relation to outcomes of importance during the last block of five sequential interviews.

### 2.3 Data collection and analysis

In‐depth interviews were conducted by HL and were conducted in participants’ homes. The interview guide used open‐ended questions, and probes were drawn from a stakeholder conference and literature review. Interviews were audio‐recorded and transcribed verbatim.

#### 2.3.1 Step 1 Analysis

The core team (HL, JL) discussed and refined themes frequently during coding. Other members of the team (RF, MP) also coded a sample of transcripts. Frameworks 17 were subsequently constructed to explore the strength of themes and the relationships of supporting themes.

#### 2.3.2 Step 2 Analysis

To gain insight into well‐being status, the interview began by asking how the participant was feeling and the factors that contributed to this. This produced a self‐assessment of well‐being that was explored during a cross‐case depth reading of each transcript. Participants were subsequently allocated to one of four well‐being groups with frameworks used to identify the pattering of themes across groups. This process involved searching through the transcript of each participant to identify language and text to support or refute their initial well‐being designation. Researchers MP and RF subsequently performed a blinded depth reading of a random sample of participant transcripts to agree or refute HL's initial categorizations.

#### 2.3.3 Step 3 Analysis

Following steps 1 and 2, participant‐defined well‐being was then used to frame more detailed analysis to identify causal relationships of a context‐mechanism‐outcome formation by working forwards and backwards through each transcript. Causal relationships were identified according to how respondents indicated causality in relation to context and outcomes during the interviews. For example, relationships between variables were plotted according to whether an individual cited them as a life context factor: “I need/I have X in place” (low‐dose meds or meds with few side‐effects), “before I achieve Y” (mechanism/short‐term outcome: peer support groups), “which will help me achieve Z” (long‐term outcome: more positive sense of self). These relationships are depicted in Figures 2‐5 which are context‐mechanism‐outcome relationships for each well‐being cluster. These configurations were built from “if then statements” extracted from the transcripts, which represent a theorizing by the participant during the interview. For this study, we conceptualized short‐term outcomes as functional mechanisms or steps towards the achievement of long‐term goals.

This approach is often used in Realist studies^18^, ^19^ that provide an exploration of how variables interact to produce mechanisms and outcomes. Specifically, they seek to understand how features of sociocultural, political, material or other contexts interact to trigger causal mechanisms and subsequent outcomes.

Analysing causal relationships according to participant‐defined well‐being facilitated the formulation of a theory of change model across well‐being groups that captured the important outcomes and the factors that shape them. It allowed us to specify for whom (participant‐defined) in what circumstances (role of life context) certain mechanisms (short‐term goals) support long‐term outcomes. This is particularly important in schizophrenia which is characterized by heterogeneity of lived experience. Importantly, this approach highlighted the often incremental nature of recovery and that well‐being and illness states are not static. Understanding outcomes according to well‐being status also permitted the amplification of the voice of those who are most unwell, which is often obscured by the voices of those who are less ill in research studies.

## 3 RESULTS

### 3.1 Participant characteristics

Table 1 provides a socio‐demographic overview of the sample. Participants’ mean age was 40 years; half of the sample was female with a similar proportion in employment (in some capacity). Less than three quarters of the sample were white British with the remainder originating from black African, British Indian and Italian. The mean duration of illness was 17 years. All except one participant were taking antipsychotic medication.

**Table 1:** Characteristics of the sample

	Participants n. 22
Age, years: mean and SD range	40 (11) 23‐60
Female: n %	11 (50)
Employment, n (%)	
Employed, full‐/part‐time	13 (60)
Unemployed/DLA or incapacity benefit	8 (36)
Student	1 (4)
Marital status, n (%)	
Married/co‐habiting	7 (32)
Divorced/separated	1 (4)
Single	14 (64)
Widowed	‐
Ethnicity, n (%)	
British, White	16 (73)
African, Black	2 (9)
British, Indian	2 (9)
Italian	2 (9)
Schizophrenia (treatment resistant)	2 (9)
Schizophrenia (simple)	14 (64)
Schizophrenia (paranoid)	3 (13.5)
Schizoaffective disorder	3 (13.5)
Duration of illness, years: mean and s.d. range	17 (9) 2‐30

### 3.2 Outcomes of importance

Participants varied in how they talked about their well‐being. This ranged from those who felt “recovered” (n=2), to those who felt they were “doing well” (n=10), others who felt that they were “Improving” (n=8) and those who felt that they were “not doing well” (n=2).

Eight long‐term outcomes that were frequently talked about and highly valued by participants: employment, a positive sense of (normal) self, reductions in symptoms, psychosocial and functional improvement, improved cognition, social connectedness, safety and security and improvements in physical health (see Table 2).

**Table 2:** Important long‐term outcomes

Outcome domain of Importance	Reported and Valued by:
Employment	All participants (except those who were recovered)
Positive Sense of (normal) Self	
Regain Self	Not doing well, Doing well
Feel confident, purposeful and responsible	Improving
Be in control, independent and dignified	Doing Well
Sense self‐worth and value	Recovered
Reductions in Symptoms	
psychosis and anxiety	Not doing well, Improving
anxiety, panic and depression	Doing Well
Psychosocial and functional Improvement	
Improve and maintain daily functioning and coping	Not doing well, Improving
Achieve normal milestones (residential independence)	Doing Well
Improved Cognition	
Improve clarity of thinking and creativity	Recovered
Social Connectedness	
Feel Socially Connected	Not doing well, Improving, Recovered
Safety and Security	
To Feel Safe and secure	Doing Well
Improvements in Physical health:	
Better diet, more exercise, better sleep, manage comorbid conditions	Not doing well, Improving

*Employment* as a long‐term outcome was valued by all participants; it was related to sense of purpose, confidence and self‐worth. The quote below describes why employment was such an important outcome for one individual—a view that resonated with other participants:

It gives me routine, social contact, real purpose, like when you're staying in for hours on end and not doing very much, it….you begin to wonder what you're doing? And wondering whether efforts that you made at school or at University are really worth anything, and social contact as well. P16

Employment was not a desired outcome for participants who considered themselves recovered because was already achieved. For these individuals, employment was part of their life context and it served to maintain their well‐being.

*A positive sense of (normal) self* was also valued across the groups and was related to employment and the achievement of life milestones such as residential independence. For those who were not doing well, an emphasis was placed on regaining a sense of self. For participants who were doing well, the emphasis was on regaining a sense of “positive/normal” self:

Well I wasn't really a teenager but I have a normal sort of young person's life, I had…I studied and I had a job and I had a social life; I could go out with friends and it was just being a normal person and having a normal life and not being seen as some freak or something. P14

These participants also wanted to regain control of their life and feel independent; this was often achieved through residential autonomy:

And there was no privacy or dignity or anything like that and I couldn't be independent even though I wanted to be independent. And [um], and then – thank god for that – I got myself a council flat. P02

For recovered individuals, a positive sense of self was achieved through achieving self‐worth and value.

*Reductions in symptoms* were important for all participants with the exception of those who felt recovered. Participants were however more often concerned with feelings of anxiety, depression and panic than positive symptoms such as hallucinations:

It's something I'd like yeah. The focus has been a lot about paranoia and just giving me a prescription for a pill to see if it would help with the anxiety but not going deep into that, it has been more about psychotic symptoms as opposed to the anxiety. P17

*Psychosocial and functional improvements* were valued by participants. For the individuals who were not doing well, the emphasis was to improve or maintain their daily functioning and coping mechanisms:

Yeah, I'm alright, I, I do alright, you know my flat's not that untidy…you know I'm actually pretty lucky, I can look after myself just, I'm on the cusp of I can do it, whether a single person living on their own can really cook, and work, and clean, I still have my doubts… P15

Those who were doing well wanted to achieve “normal milestones” such as residential autonomy and independence.

*Improved cognition* was particularly valued as outcome for the recovered participants, and for one individual, his cognition improved when he stopped his medication:

[um] And I think really what brought me back was first of all stopping the medication, OK so I could think better, more clearly. P13

*Social connectedness* was valued by the majority of the sample either as a short or long‐term outcome:

I guess like the social interaction really, I've been single for quite a long time, I don't go out and see my friends so much because effects of….well most of them quite a few of them have got families and they're all doing their own … P16

*To feel safe and secure* was important to the doing well group, and this was independent of talk related to stabilizing medication, which was articulated by the rest of the sample as a way to achieve this:

Somewhere to feel a bit safe in a way, from all the stress. P15

*Improvements in physical health* such as a better diet, more exercise, better sleep and management of co‐morbid conditions was important for the majority of the sample:

[um] Well like I said I had Hepatitis C and I've had cancer …I am overweight and yet I eat very little sugar and very little fat and still I am overweight and I've only recently discovered that one of the secondary effect of the medication. P05

### 3.3 The relationship between life context, participant‐defined well‐being and the achievement of short‐term goals and long‐term outcomes

**Figure 1:**
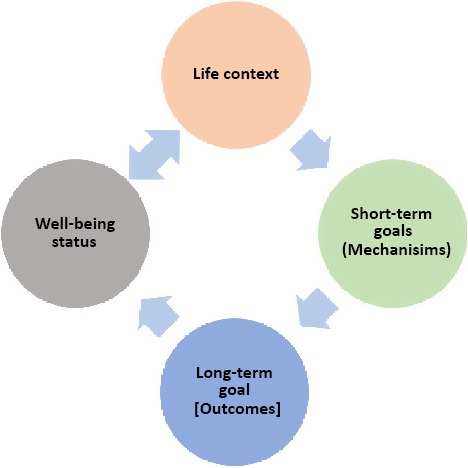
The circular relationship between life context, short‐ and long‐term outcomes and well‐being

Our findings suggest a theoretical change model where features of life context influence the attainment of long‐term outcomes through short term goals (mechanisms) (see Figure 1). Participants also indicated that there was a reinforcing and interactive relationship between well‐being and life context. For example, improvements in life context would also support enhancements in well‐being and vice versa. Features of life context that influence well‐being in this sample were appropriate treatment (CBT, the presence and absence of medication), familial support, employment and meaningful activities and faith. Most notably, participants in this study held a strong consensus that for medication to be useful (eg for it to have a stabilizing impact upon symptoms and mood), side‐effects must be minimal and well controlled. When this had been achieved, participants reported that medication was able to provide a stable basis for attainment of short‐term goals (mechanisms) such as social interaction and meaningful activity.

**Figure 2:**
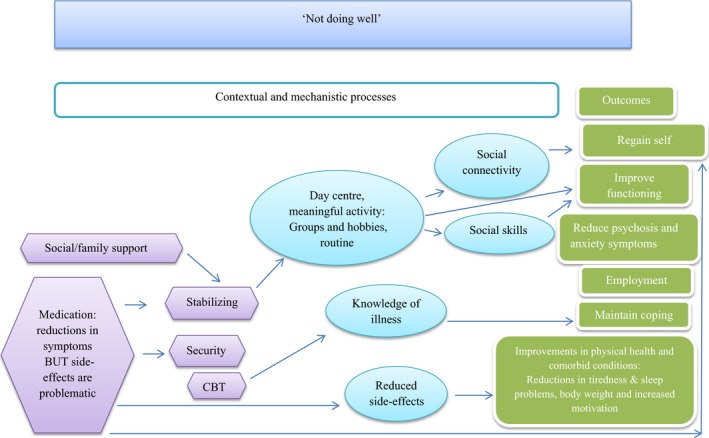
Not doing well: the relationships between life context, mechanisms and outcomes

**Figure 3:**
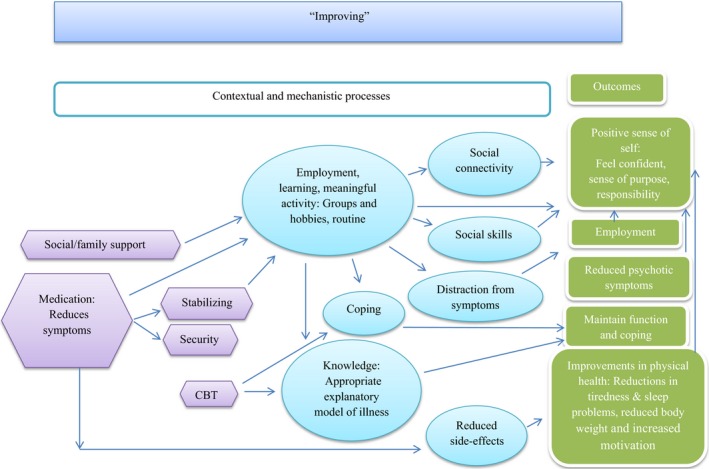
“Improving”: the relationships between life context, mechanisms and outcomes

**Figure 4:**
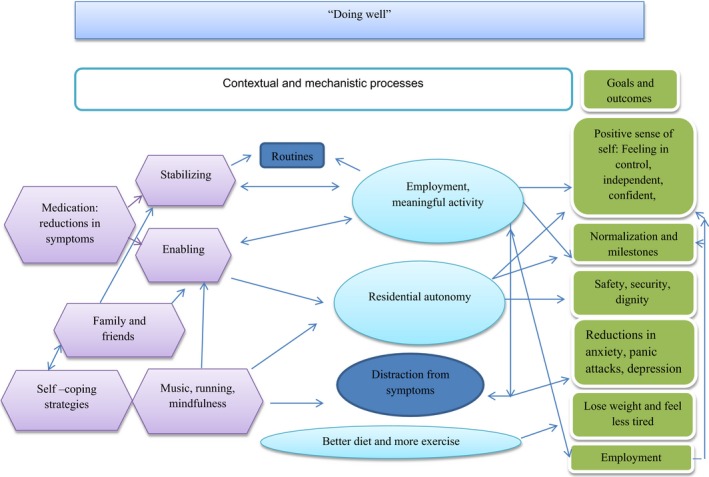
“Doing well”: the relationships between life context, mechanisms and outcomes

**Figure 5:**
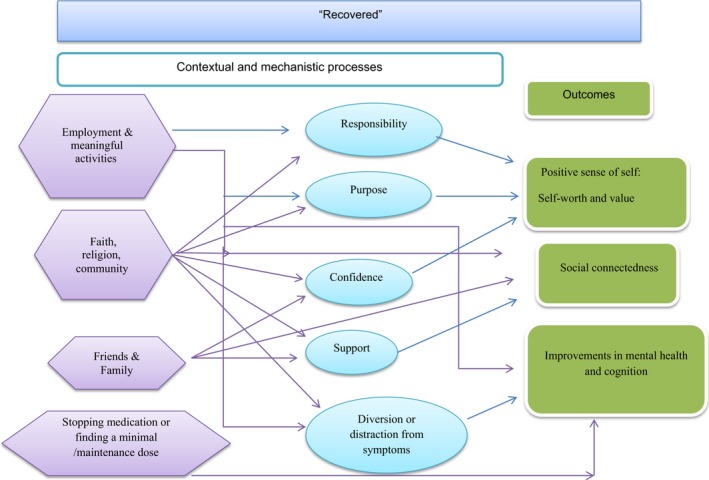
“Recovered”: the relationships between life context, mechanisms and outcomes

Secondly, strong inductive themes (those presented in Table 2) and observed relationships among them support the notion that participant‐defined well‐being influences the type of short‐term goals (mechanisms) and long‐term outcomes that individuals could envisage. This in turn was also influenced by an individual's life context. This circularity was detected by analysing the sample as a whole and then by well‐being cluster. The latter also provided an opportunity to distinguish and unpick the broad domains of outcomes into more specific and more personalized subdomains. This also revealed a change model which suggests that as an individual's well‐being improves or declines, this influences the short‐term goals (mechanisms) and long‐term outcomes that are possible, and the contextual features that support or impede them. To demonstrate this, each well‐being group is described below with the causal relationships that they felt operated between life context and their desired outcomes. Figures 2, 3, 4, 5 depict the context‐mechanism‐outcome formations described by the participants.^2^

#### 3.3.1 Not doing well

Two participants described themselves as “not doing well.” Figure 2 presents the relationship between life context and short‐term goals (mechanisms) with long‐term outcomes as articulated by these individuals.

These participants desired improvements to their physical and mental health, the maintenance of coping and desired improvements in social functioning. These participants felt stuck, and they placed an importance on regaining a sense of self. To achieve this, they felt that they needed to be more socially connected by attending a day centre or through hobbies and other meaningful activities. These short‐term goals/mechanisms act as a way in which to increase social skills through interactions with others, but also provide an opportunity to rebuild identity through a reconnection or discovery of people, places and activities of value. They also believed that these activities help improve daily functioning through skills acquisition and/or an improved sense of self‐efficacy. The following quotes describe how use of a personal budget has helped this participant achieve these aims by attending a computer literacy course:

How? [um] Well one, it fills my time up; two, I get to meet people in a similar situation to myself. Like I say, safe environment. The guy doing the instruction ‐ – very empathetic. OK I'm learning to integrate up there ‐ what else? Useful skills. P10

Reductions in symptoms of psychosis and anxiety were also desired outcomes articulated by these individuals, but they were unable to identify mechanisms to achieve this. Both individuals also desired employment, but this was a long‐term goal.

The life context of these individuals was characterized by minimal social/familial support, and they viewed medication as a stabilizing function to provide a sense of security, but unfortunately, both individuals reported problematic side‐effects.

#### 3.3.2 Improving

For the eight individuals who considered their well‐being to be improving, employment and participation in structured activities were ways in which to achieve a positive sense of self. Their narratives where characterized by momentum; they were able to articulate outcomes with a sense that these were achievable through short‐term goals (see Figure 3).

For these individuals, a distraction from symptoms was achieved through employment and meaningful activities as evidenced by the following quote:

So that could be giving back but I think it helps like I said – when I'm learning it helps me stabilise, it helps my brain to stabilise because if I stay in one place, it doesn't help me, so many negative thoughts, but when you are busy, you know, it keeps…your brain occupied. P08

For this group, there was a more positive sense of the contribution of medication as a basis for achieving outcomes. This was due to the fact that side‐effects were less of a problem than in the “not doing well” group.

I'd say, mentally I'm doing a lot better on abilify, just being more me, happier, my confidence is still not quite that high though... P017

#### 3.3.3 Doing well

Ten participants felt that they were doing well, and six were working in full‐time professional jobs:

Well I feel I'm doing pretty well at the moment, I've been symptom free for a couple of years and I've got a good job, perhaps not as independent as I could be but I'm happy with the way things are at the moment, I keep physically fit, I could be more sociable, I have had problems with anxiety, yeah pretty good for the last couple of years. P16

There were however feelings of frustration among some of these individuals, which was often related to not having reached life milestones, in particular paid employment. The articulation of employment as a life milestone is a distinguishing feature between this group, and the two previous groups, suggesting that these participants had higher expectations and increased frustration when these were not met. This suggests that enhanced well‐being increases the expectations participants have in relation to long‐term outcomes (see Figure 4).

Among this group, a positive sense of self was related to a need to feel in control of one's life. This was coupled with an importance attached to being independent and confident, described by the following individual who undertook a gap year after completing his degree course:

Yeah, I was on that for a while and then [um] I went travelling, that's why I went on the tablets [um]. Probably wasn't [um]… advised by my carers but, you know, I wanted to go away, I wanted a break from everything so I went away. P07

Residential autonomy was also a way to achieve a positive sense of self for this group. The life context features which supported the achievement of outcomes were similar to those of the “improving” group, particularly the importance of medication as stabilizing. This enabled the achievement of employment and residential autonomy.

Just because I wanted to do it but I just couldn't do it sort of thing. [um] Then I went on this medication and I could do it, I could do all the things I wanted to do. P14

Other features of life context, such as self‐coping, exercise, mindfulness contributed to long‐term outcomes. Interestingly, the emphasis on self‐coping as a life context among this group contrasts with references to coping in the “not doing well” and “improving” groups, suggesting an increased sense of self‐efficacy. The shift to self‐coping suggests a change that occurs when individuals start to feel that they are doing well, that is with the utilization of strategies of support at this stage of well‐being. The use of meditation is described in the following quote as such a strategy:

Yeah, yeah like a safety mechanism sort of thing. Because I mean, yeah it's really revolutionised the way I've…I feel a lot happier you know, it really has helped [um]…yeah it has helped. P07

#### 3.3.4 Recovered

Two respondents considered that they were recovered from schizophrenia—both were employed and were living productive lives. One individual had been medication‐free for a number of years without relapse. The factors associated with a positive sense of self voiced by the other groups, such as feeling independent and in control, were not articulated by these individuals; this may be a reflection of the sense of personal agency and efficacy both felt as a consequence of feeling recovered. For example, having responsibility and purpose in life (e.g. as a parent) contributed to a sense of self‐worth and value‐providing features of life context were in place such as employment (see Figure 5).

The shift in employment from an outcome (as in the other groups) to a life context here suggests that perhaps once achieved, employment ceases to be an outcome and becomes a stabilizing force in people's lives and provides further support for the notion that outcomes change in emphasis in relation to well‐being:

I mean a sense of purpose, you know, that you are of value, that you are important, that, you know, what you have to say to people does matter…. something purposeful I'd say, purpose... P11

Social support was highly valued as mechanism to increase self‐confidence and feelings of social connection, and achieve recovery‐focused outcomes among this group:

You know I've been singing in the choir since about 2002. And [um] singing really helps. You know people together… I've been able to almost become a sort of leader within the choir, so that gives me confidence ….. standing in front of a hundred people is powerful, confidence again. It's such a simple thing. P13

Reducing or stopping medication was perceived as a feature of life context that individuals attributed to enhanced cognitive abilities such as increased creativity. Both felt that medication dampened these functions. The diversion from symptoms afforded by work or engaging in meaningful activities also provided a way to help increase clarity in thinking and creativity. For one participant, maintaining a minimum dose Olanzapine was a worthwhile trade‐off between the need for creativity and stability:

And in one way I'd like to…oh no I won't say it…if I were to stop taking my [er] tablet probably I'll have more creativity but then I wouldn't…I'd be haywire so I wouldn't do that anyway. P11

### 3.4 Stability and change in outcomes of importance across well‐being groups

**Figure 6:**
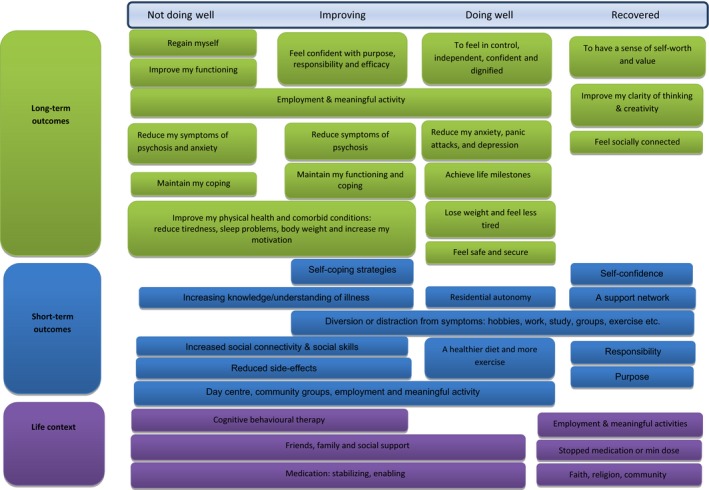
Life context, Short‐ and Long‐Term Outcomes by Participant‐Defined Well‐being

Our model suggests that there are outcomes that are stable across well‐being groups (employment, positive sense of self), while others may change and develop. This is demonstrated in the elaboration of the model depicted in Figure 6. This model was built by combining the context‐mechanism‐outcome configurations for each well‐being cluster. For example, for those who were not doing well, exercise was not a short‐term outcome/mechanism, whereas this serves as a mechanism for the other groups. Likewise, while employment is an outcome desired by the majority of the sample, for the recovered group, it becomes a feature of their life context. At a deeper level, it is also apparent that subtypes of higher order domains have the potential to subtly change over time in relation to life context and well‐being, for example “to feel confident” in the improving group to “feeling in control” in the doing well group (see Figure 6).

## 4 DISCUSSION

Our study explored the outcomes that matter for people with schizophrenia and the mechanisms that shape their attainment by self‐defined well‐being status. Broadly speaking, these outcomes were achieving life milestones, feeling safe, being socially connected, having a positive sense of self, reductions in symptoms and outcomes related to improved physical health. The majority of the sample valued these outcomes, which were also identified as important by family members that we interviewed for our carer study.^20^

The importance of achieving participant‐defined life milestones was an outcome evidenced by the value placed on specific psychosocial and functional/occupational activities such as gaining employment, completing university, achieving residential autonomy or getting married. There was a sense of the importance of these outcomes across both the patient and carer samples.^20^ Achieving life milestones was also intertwined with and supported by the direct achievement of other short‐term outcomes such as those related to coping and reducing symptoms. Attaining a positive sense of self by increasing control, confidence and a sense of value and purpose, and increasing social connectivity were also facilitative to achieving life milestones.

Individuals also valued feeling safe and secure. For those who were doing well, this was related to having their own accommodation, suggesting an externalized notion of risk from the outside world. For those who were doing less well, safety as an outcome was articulated as an internal feeling brought about through the stabilizing effects of medication that is a sense of safety by the reduction of symptom behaviours. This notion of safety was more aligned to that expressed by the carers we interviewed,^20^ who perceived risk to the individual or others as a consequence of symptoms of illness.

Recent policy has highlighted the importance of physical health outcomes for people with severe mental illness calling for a “parity of esteem”.^21^ Our research confirms that people with schizophrenia also value physical health. Low levels of exercise, poor diet and increased body weight were concerns among this sample that were also reflected in other studies.^22^, ^23^ Participants described weight gain as resulting from the side‐effects of medication, and difficulties engaging in exercise due to amotivation were commonly voiced. The fact that this was raised as an outcome has the potential for better engagement of people with schizophrenia with managing their physical health; although as previous research has articulated, this needs to be facilitated by adequate access to general practice and professionals who can monitor and support goals relating to physical as well as mental health.^24^

Helping individuals to achieve better outcomes will require an understanding of the context of their life and the features of this that need to be in place to achieve short‐ and long‐term outcomes. Our model suggests that well‐being status has the potential to determine what and when short‐ and long‐term goals are appropriate, but also how individuals will engage with the supporting processes and resources available to them (both statutory and non‐statutory). This presents a unique model for structuring clinical support and contact with applicability across diagnostic groups and a way in which to enact person‐centred care.

With the exception of quality of life and satisfaction with treatment, the participants in this study endorsed the broad outcome domains previously identified in the literature (e.g. symptom‐related outcomes (40); functional outcomes;^26^ positive sense of self^27^, ^28^). Subdomains of quality of life such as motivation and energy^29^, ^30^ and stigma^31^ were intricately woven and talked about in relation to other outcomes such as employment. Satisfaction with medication^32^, ^33^ was perceived as necessary for the achievement of person‐centred outcomes. Reductions in symptoms^34^, ^35^, ^36^ were also valued by our sample and their carers.^20^ However, participants desired reductions in affective symptoms, most notably anxiety. This perhaps reflects their experience that affective symptoms and experiences of social anxiety were most likely to hinder the achievement of outcomes. Of the known functional outcomes cited in the literature,^26^, ^37^, ^38^ employment and independence were most valued by our sample and were linked to life milestones and social relationships. Of the personal recovery outcomes documented in the literature, developing a positive sense of self^27^, ^28^ seemed the most important for our participants; for those who were not doing well, this regaining a sense of familiar self was most important.

The potential interaction between short‐term goals (mechanisms) and long‐term outcomes in relation to well‐being provides a deeper and more nuanced understanding of outcomes from the perspectives of people with schizophrenia. This suggests that within a theories of change framework (see Figure 6), what people value as important outcomes may appear to stay stable for some, but that as their health improves or deteriorates, there may also be subtle changes that will only be revealed through careful and sensitive communication. This will facilitate an understanding of what the sensible next steps are for a person (e.g. short term goals) and may help improve and align stepped care to recovery‐based principles. For example, when working to help someone who is “not doing so well,” reconnecting them with a sense of identity through meaningful activities of their choice might be a logical first step. Thinking about outcomes for the next stage of well‐being might involve considering how to support someone to feel purposeful, confident and responsible either through voluntary work or community activity. A further example is found in the strength by which functional outcomes were valued (i.e. employment and meaningful activity, independence and achieving milestones), and in particular, the interdependence of this with the other key outcomes such as developing a positive sense of self and controlling symptoms. This suggests that employment and meaningful activity are, depending on an individual's well‐being, either a potent short‐term goal (mechanism) towards recovery‐focused outcomes and/or highly valued long‐term goals in their own right. Our findings therefore add a deeper understanding of the ways in which the context of someone's life facilitates or hinders short‐term goals and long‐term outcomes. Working with individuals to understand their treatment goals and what they need to be in place to achieve person centred outcomes has the potential to improve therapeutic relationships and patient self efficacy.

### 4.1 Strengths and weaknesses

This qualitative study provides a person‐centred perspective and amplifies the views of people with schizophrenia. We argue that this conceptual model has analytic generalizability beyond the present study, namely how outcomes are influenced by the life context and well‐being of an individual. The analysis for this study is grounded in the experiences of those interviewed and rigorous in application. However, the experiences of those individuals who considered themselves as unwell and recovered in this study are based on a very small sample size. While this paper is not suggesting the findings of this study have generalizability, we do feel this deserves highlighting as an area worthy of further investigation with a larger sample.

## DECLARATION OF INTEREST

None.

## Notes

1 American Psychological Association: http://www.apa.org/monitor/2012/01/recovery-principles.aspx

2 Hexagons represent context, the ellipses represent mechanisms and the rectangles represent outcomes.
